# Regulation of non-emotional memory through α_1_-adrenergic receptors activation: A short review

**DOI:** 10.1016/j.ibneur.2025.02.005

**Published:** 2025-02-14

**Authors:** Eugénia Correia de Barros

**Affiliations:** Department of Neurological Sciences and Mental Health, Faculty of Medicine, Agostinho Neto University, University of Lisbon, Lisboa, Portugal

**Keywords:** α_1_-adrenergic receptors, Non-emotional memory, Long-term potentiation, Alzheimer's disease, Spatial memory

## Abstract

The α_1_ adrenergic receptors (α_1_-ARs) play a central role in the regulation of synaptic plasticity and memory, but their role in non-emotional memory is still poorly understood. This review summarizes recent advances in understanding the functions of α_1_-ARs and highlights their contributions to synaptic efficacy, long-term potentiation (LTP), and long-term depression (LTD) in the hippocampus and neocortex. There is evidence that α_1_-AR activation occurs through intracellular pathways such as Gq-protein signaling, MAPK, and cAMP cascades. Furthermore, α_1_-ARs are emerging as promising therapeutic targets in neurodegenerative diseases, including Alzheimer’s disease (AD), due to their capability to modulate cognition and neuronal plasticity. New insights into positive allosteric modulators (PAMs) that cross the blood-brain barrier provide a potential avenue for safer and more effective therapies. This review highlights the need for further research to improve the understanding of α_1_-ARs and their potential for memory enhancement and neuroprotection.

## Introduction

One of the most central questions in neuroscience has been why it is easier to remember bad things compared to good ones. Based on consciousness, memory systems are commonly classified into declarative and non-declarative. While the declarative system involves the conscious recall of facts or events mainly depending on the integrity of the hippocampus and neocortex, the latter most notable in the prefrontal cortex (PFC), in the non-declarative, information is learned and retrieved without conscious effort, mainly through the amygdala. On that premise, non-emotional memory usually emphasizes the declarative type. Many existing studies in the broader literature have observed that the consolidation of daily memories may be associated with the activation of *locus coeruleus* (LoC), a nucleus located within the dorsal wall of the rostral pons that serves as the major source of norepinephrine (Giorgi et al., 2017). Fundamentally, the action of norepinephrine on memory mechanisms is determined by the expression patterns of the various adrenergic receptor subtypes, which display similar binding affinities but evoke different physiological effects (Norman and Henry, 2015). The α_1_ adrenergic receptors (ARs) subtypes belong to the family of ARs, which also contain α_2_ and β-AR subtypes, with each subtype regulating distinct but commonly overlapping functions throughout the sympathetic nervous system. Besides their roles in smooth muscle contraction and heart modulation, some authors have suggested that increased memory for emotional experiences predominantly involves the activation of the β-adrenergic system, while non-emotional memories are mainly regulated by α_1_-adrenergic receptors (α_1_-ARs) (Noorani et al., 2020, Perez, 2021). Therefore, this short review aims to synthesize previously published studies and develop an updated overview that systematically describes the role of the α_1_-adrenergic receptor in regulating synaptic efficacy, particularly in non-emotional memory. Additionally, it will explore the potential of targeting these receptors as cognitive and therapeutic enhancers in neurodegenerative disorders.

## Overview of α_1_-adrenergic receptors

A series of recent studies has indicated that adrenergic receptors have a total of three families (α_1,_ α_2,_ β) and nine subtypes (α_1A_, α_1B_, α_1D_, α_2A_, α_2B_, α_2 C_, β_1_, β_2_, and β_3_) that, despite sharing similar affinities, offer different physiological effects for the same endogenous catecholamines (Perez, 2020). Upon further investigation using a transgenic-tagged approach, α_1A_ and α_1B_-ARs were found to exhibit similar expression patterns in the central nervous system (CNS), with distinct relative abundance in the hippocampus, as well as in areas of the cerebral cortex, hypothalamus, midbrain, and spinal cord (Papay et al., 2006).

The α_1A_-AR subtype expresses its highest density in the CA1, CA3, and dentate gyrus of the mouse hippocampus and hypothalamus, while the α_1B_-AR is more highly expressed in the cerebral cortex. Moreover, as it was previously shown *in vivo*, the α_1A_-AR subtype is expressed in key cognitive centers of the brain, and its agonists’ activation can increase cognition, synaptic plasticity, and long-term potentiation in normal wild-type mice (Papay et al., 2023). In the brain, norepinephrine (NE) is synthesized from dopamine hydroxylation in noradrenergic neurons, primarily in the *locus coeruleus,* transported by vesicular monoamine transporters (VMAT) into synaptic vesicles, released into the synaptic cleft, then finally binds to adrenergic receptors on postsynaptic or presynaptic neurons. To avoid overactivation, norepinephrine is either reabsorbed into the presynaptic neurons via the norepinephrine transporter (NET) or degraded by monoamine oxidase (MAO) and catechol-O-methyltransferase (COMT). At this stage of understanding, both α_1_-AR subtypes represent a crucial role in regulating short and long-term synaptic plasticity, and different types of memory, mostly the declarative system (Perez, 2020).

## Transduction and signaling pathways

After its release, α_1-_ARs couple to the heterotrimeric G_q_ (Gαq) family of G-proteins to activate phospholipase C (PLC), which leads to the breakdown of membrane-bound phosphatidylinositol 4,5-bisphosphate (PIP_2_) into inositol triphosphate (IP_3_) and diacylglycerol (DAG). The calcium channel opening is regulated by the IP_3_, resulting in Ca^2 +^ release from the endoplasmic reticulum, and the DAG activates protein kinase C (PKC), which modulates various cellular proteins (Perez, 2021). Although it has not been shown *in vivo,* several reports have demonstrated that α_1_-ARs couple to Gi G-proteins under overexpressed conditions (Cotecchia, 2010). Likewise, α_1-_ARs are able to signal through independent mechanisms that involve β-*arrestins* that represent a scaffold and allosteric activator for protein kinase signaling cascades, amplifying these pathways' activity within the cells (Liu et al., 2024). Another crucial signaling pathway is through Mitogen-Activated Protein Kinase (MAPK), which influences cell proliferation, differentiation, and survival. Particularly, this pathway has been linked to neuroplasticity and long-term cellular changes, being relevant for memory processes and a potentially good target to treat a wide variety of neurodegenerative conditions with impaired cognition (Buzsáki, 1989, Maity et al., 2020).

## α_1_-AR activation, synaptic plasticity, and non-emotional memory

The CA3 pyramidal neurons are a specific type of neuron in the hippocampus that generates rhythmic activity with the CA1 field. Prior research has demonstrated that when α_1_-ARs are activated, the rhythmic spontaneous electrical discharges between both areas can decrease or stop, altering the downstream signaling. For instance, this synchronized activity is often associated with long-term potentiation (LTP), enhancing the neurons’ ability to communicate and contribute to memory formation and learning over a long period (Buzsáki, 1989).

Hence, the fact that the strength of synaptic transmission was enhanced by the activation of adrenergic receptors via cAMP signals, several studies had concluded that the only adrenergic receptor that mediated NE effects on long-term plasticity was β-AR (Maity et al., 2020, Nguyen and Gelinas, 2018). However, this was inconsistent with previous studies, where α_1-_ARs had been shown to mediate increased cAMP signaling independent of β-AR effects (Stone et al., 1987, Huang and Daly, 1972). These findings align with studies showing that α_1_-ARs contribute to synaptic plasticity in the neocortex by mediating glial-neuron interactions. Specifically, α_1_-AR activation stimulates astrocytes, prompting the release of ATP, activating purinergic receptors on pyramidal neurons. This cascade ultimately enhances long-term potentiation (LTP), a key mechanism for memory and learning (Wahis and Holt, 2021, Pankratov and Lalo, 2015, Man et al., 2023). Equally important, in long-term depression (LTD) as the opposite of LTP, α_1_-ARs have been shown to induce it at CA3-CA1 synapses in the rat hippocampus, despite significant alterations in noradrenergic input (Dyer-Reaves et al., 2019). Although there is no evidence of which α_1-_AR subtype mediates, these findings support the notion that both mechanisms of LTP and LTD may be similar partners in the synapse flexibility to store information (Heynen et al., 1996).

Several experimental studies are reported in the literature to address the role of α_1-_AR in non-emotional memory. A recent study that used transgenic mice engineered to express a constitutively active mutant (CAM) form of the α_1A_-AR and normal mice treated with an α_1A_-AR-selective agonist, cirazoline, revealed its significance in several behavioral models of memory, as well as increased synaptic plasticity and long-term potentiation. In contrast, α_1A_-AR KO mice displayed poor cognitive function (Va et al., 2011). In addition, concerning positive allosteric modulators (PAMs), studies have demonstrated that PAMs, which can cross the blood-brain barrier, enhance α_1_-AR function in the CNS, playing a pivotal role in improving cognitive functions. This involves norepinephrine (NE)-mediated cAMP signaling, where α_1_-AR activation increases intracellular cAMP levels, facilitating memory-related processes through downstream pathways such as PKA and CREB activation (Kandel, 2012, Kida and Serita, 2014).

## Spatial memory

In addition to non-emotional memory, the hippocampus also regulates spatial and associative learning functions. This has been explored in prior studies using the Morris water maze test by Puumala *et al.* on the interaction between α_1_-AR and muscarinic cholinergic systems upon the regulation of spatial navigation behavior, indicating that the stimulation of α_1_-AR in the CA1 region of the hippocampus may facilitate the encoding of new information (Puumala et al., 1998). A similar pattern of results was obtained in the following studies, where the transgenic mice that overexpressed a constitutively active α_1A_-AR improved spatial memory in the Barnes, Morris, and multi-T type mazes (Doze et al., 2011, Mishima et al., 2004).

Moreover, the concept of working memory lies in the retention of a small amount of information in a readily accessible form. Consequently, spatial working memory involves the ability to keep spatial information active in working memory over a short period, relying more on the prefrontal cortex than the hippocampus. A substantial body of research suggests that α_1_-AR activation enhances working memory by promoting focus and attention (Hvoslef-Eide et al., 2015). Additionally, the wake-promoting effects of a single dose of modafinil are thought to involve α_1_-ARs, as prazosin – a selective α_1_-AR antagonist – has been shown to counteract modafinil-induced improvements in execute cognition (Winder-Rhodes et al., 2010). Also, α_1_-ARs have been demonstrated to regulate this sort of memory through the release of glutamate in the PFC due to a sustained excitatory effect on the pyramidal neurons, yet with opposite effects when excessive stimulation, such as stress exposure occurs (Datta et al., 2019, Yan and Rein, 2022). Interestingly, besides promoting mesolimbic transmission in the medial PFC, α_1_-ARs are expressed on presynaptic terminals in the nucleus accumbens, where they regulate dopamine (DA) pathways. These findings may support the notion that with GABAergic regulation, these networks offer a significant role in modulating reward-related memories (Solecki et al., 2022).

## Therapeutic implications and future directions

The LoC neurons constitute the main neurons that are affected in Alzheimer’s disease (AD) with a loss of around 70 % in affected patients (Bekdash, 2021), which may suggest that the use of NE reuptake inhibitors could be a selective treatment in early stages of AD. Historically, β-ARs were deemed safer than α_1_-ARs due to the latter’s association with cardiovascular side effects, including hypertension, tachycardia, and arrhythmias. These adverse effects result from α_1_-AR-induced vasoconstriction and increased cardiac workload, which are less prominent with β-AR activation. However, despite the side effects and the poor brain penetration for most of the current α_1_-ARs agonists in neurological conditions, a new PAM with high selectivity for the α_1_-AR subtype has been developed. This PAM is able to cross the blood-brain barrier (BBB) sufficiently enough to improve cognitive functions and modify Alzheimer’s mouse models without increasing blood pressure (Papay et al., 2023).

In post-mortem tissues from patients with Alzheimer’s disease (AD) and dementia with Lewy bodies (DLB), despite the loss of neurons, α_1_-AR binding sites were either maintained or increased in the PFC and hippocampus, which could reflect a form of neuronal plasticity through a compensating mechanism (Szot et al., 2007). These findings tie well with the previous studies wherein to study the interaction between amyloid-β (Aβ) and tau and their effect on synaptic function, the 3xTg-AD mouse model that contains three genetic mutations associated with familial AD (APP Swedish, MAPT P301L, and PSEN1 M146V), when given a selective α_1_-AR positive allosteric modulator, spatial memory was improved along with LTP. This approach provides a valuable model for evaluating potential AD therapeutics (Oddo et al., 2003, Zhang et al., 2023).

## Conclusion

On this basis, this review has developed an updated overview that systematically describes the role of the α_1_-adrenergic receptor in regulating synaptic efficacy in non-emotional memory and has explored the potential of targeting these receptors as cognitive and therapeutic enhancers in neurodegenerative disorders, such as Alzheimer’s and Lewy bodies disease. Looking forward, further research should aim to explore these aspects in greater depth, offering more experimental approaches, not only from a therapeutical perspective but also for enhancing memory processes in healthy individuals.

## Declaration of Competing Interest

The authors declare that they have no conflicts of interest that could influence the interpretation or presentation of the research findings. Any financial, personal, or professional relationships that might be perceived as potential conflicts of interest are disclosed.
